# Mechanistic Insights into Protein Corona Formation: The Surface Charge of Mesoporous Silica Nanoparticles Determines the Orientation and the Conformation of Adsorbed BSA Protein

**DOI:** 10.1021/acs.langmuir.5c06171

**Published:** 2026-03-27

**Authors:** Alessandra Ballicu, Gaia M. Meloni, Matteo Farci, Davide Tocco, Marco Piludu, Drew F. Parsons, Cristina Carucci, Barbara Jachimska, Andrea Salis

**Affiliations:** a Department of Chemical and Geological Sciences, 3111 University of Cagliari, Cittadella Universitaria, S.S. 554 bivio Sestu, Monserrato (CA) 09042, Italy; b Center for Colloid and Surface Science, via della Lastruccia 3, Sesto Fiorentino (FI) I-50019, Italy; c Department of Biomedical Sciences, 3111 University of Cagliari, Cittadella Universitaria, S.S. 554 bivio Sestu, Monserrato (CA) 09042, Italy; d 132074 Jerzy Haber Institute of Catalysis and Surface Chemistry Polish Academy of Sciences, Krakow 30-239, Poland

## Abstract

The formation of the protein corona critically governs the biological identity of nanoparticles, but the molecular determinants of protein orientation and conformational fate remain elusive. Here, we examine the adsorption of bovine serum albumin (BSA), a prototypical dysopsonin, onto bare and amino-functionalized mesoporous silica nanoparticles (MSN and MSN-NH_2_) at physiological pH. Zeta potential titrations, quartz crystal microbalance with dissipation monitoring (QCM-D), and circular dichroism (CD) spectroscopy reveal robust binding of BSA to both negatively charged MSN and positively charged MSN-NH_2_. QCM-D quantification indicates enhanced adsorption on MSN-NH_2_ (481 ng cm^–2^) relative to that on MSN (228 ng cm^–2^), consistent with attractive electrostatic interactions. Strikingly, substantial BSA adsorption also occurs on MSN, even though both species carry negative zeta potentials. This indicates orientation-dependent interactions driven by the heterogeneous charge distribution of BSA, likely involving domain III binding to the silica surface. CD spectroscopy further demonstrates that the nanoparticle surface charge dictates the BSA secondary structure: the α-helix content decreases from 60% to 25%, while β-sheet and random coil fractions increase upon adsorption to MSN-NH_2_. Whereas BSA retains its native (N) conformation on MSN, it undergoes pronounced distortion toward fast (F) or elongated (E) states on MSN-NH_2_. These findings establish that nanoparticle surface charge governs not only the adsorption extent but also protein orientation and conformational fate, thereby shaping protein corona formation and its downstream biological identity.

## Graphical abstract

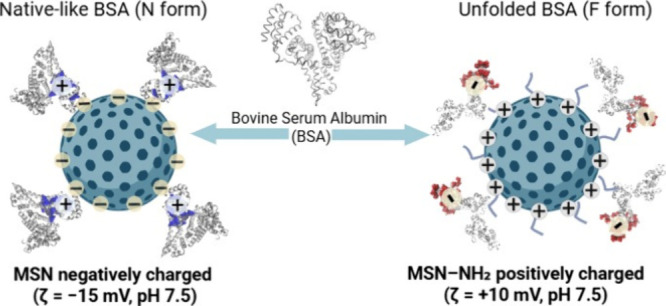

## Introduction

Nanoparticle-based drug delivery systems are of extreme importance due to their potential ability to overcome biological barriers and to go directly to the target of interest with the aim of reducing side effects.^1^ However, nanoparticles in contact with biological fluids are subject to the formation of a so-called “protein corona”.^2^ The proteins adsorbed on the surface of nanoparticles modify their physicochemical properties and alter their behavior, including colloidal stability, cell uptake, targeting, endocytosis, and pharmacokinetics.^3,4^ Depending on the binding affinity with the nanoparticle surface, proteins may form a strongly bound “hard corona” coexisting with a more dynamic and weakly associated “soft corona”. Irreversibly bound proteins constitute the hard corona, whereas the soft corona is constituted of proteins that establish weak and reversible interactions with the surface of nanoparticles.^5^ Proteins constituting the protein corona can be classified as opsonins (e.g., fibrinogen and IgG) if they promote the rapid clearance of nanoparticles from blood or dysopsonins (e.g., albumin and apolipoproteins)^6^ that prolong their circulation in blood. Hence, the binding of dysopsonins on nanoparticles can enhance the bioavailability of nanosized drug delivery systems. To do that and to understand the fate of nanoparticles under biological conditions, it is essential to investigate how to control dysopsonin adsorption on nanoparticles. Bovine serum albumin (BSA) is a dysopsonin globular protein of 66.5 kDa^7^ with an isoelectric point (IEP) in the pH range 4.7–4.9 at 25 °C.^8^ Depending on pH, the BSA structural conformation changes from a native (N) form at physiological pH to a fast (F) or elongated (E) form at low pH. The hydrodynamic radius of BSA is typically 3.5–4.0 nm in the N form from pH 4 to pH 9, while it can significantly increase to 6.0–8.0 nm in the F or E forms.^9,10^ BSA adsorption has been proven to enhance the blood circulation time of silica nanoparticles.^11^ The formation of the protein corona can be considered as a complex phenomenon resulting from the interplay of several forces, such as electrostatic,^12^ van der Waals,^13^ and hydrophobic^14^ interactions. The specific interaction primarily responsible for the formation of the protein corona varies from case to case, with the hydrophilic–hydrophobic nature of the nanoparticles playing a key role. Rae and Jachimska studied the formation of complexes between BSA and G5.5 dendrimers exploiting how protein–nanocarrier interactions depend on BSA and G5.5 surface potentials.^15^ Moreover, the distortion of the BSA secondary structure increased by increasing the molar ratio between G5.5 and BSA, suggesting a strong electrostatic interaction between the positively charged dendrimer and the negatively charged BSA at the investigated pH of 7.5. Baler et al. demonstrated how electrostatic interactions promote significant distortion of the BSA secondary structure resulting in a partial denaturation.^16^ Gu et al. measured how the van der Waals energy, together with a contribution of hydrophobic forces, was the driving force for BSA adsorption onto carbon-based materials.^17^ The adsorption of albumin on polyvinyl chloride was also found to be due mainly to van der Waals interactions.^17^ We recently proposed that BSA adsorption on aminopropyl-functionalized mesoporous silica nanoparticles (MSN-NH_2_) is mainly determined by the interplay between the electrical double layer (EDL) and van der Waals (vdW) interactions.^18^ The importance of the former was demonstrated by the modulation of the amount of adsorbed protein obtained by systematically changing the type of buffer used to regulate the pH, which was fixed at 7.15 for all experiments. Lastly, long-range noncovalent hydrophobic interactions may influence the protein corona formation, with some proteins tending to adsorb to a high extent onto hydrophobic surfaces.^19–21^ Kreuter et al. studied the adsorption of apolipoprotein E on poly­(lactic-*co*-glycolic acid) (PLGA) nanoparticles showing an important adsorbed amount of proteins onto hydrophobic nanoparticles.^22^ Mesoporous silica nanoparticles (MSNs) are essential carriers for drug delivery.^23–27^ They are typically characterized by a particle size in the range of 50–200 nm and high surface areas, typically 600–800 m^2^/g and beyond.^28^ Nanoparticle size is a critical parameter as it strongly influences interactions with lipid membranes, cellular uptake pathways, and intracellular fate. Nanoparticles with diameters below 200 nm are generally considered more relevant for membrane interaction and endocytic uptake.^29,30^ MSNs present very high thermal and chemical stability and easy functionalization and can reach high loadings of drug molecules in their pores. Among MSNs, MCM-41 (Mobil Composition of Matter n. 41) has pores that run along the entire nanoparticle length creating parallel channels arranged into a hexagonal phase.^24,25^ In biological systems, MSNs do not necessarily persist intact but are processed through cellular uptake, with surface functionalization modulating clearance and kinetics.^31^ Protein conformational changes upon adsorption onto nanoparticles are strongly influenced by the nanoparticle physical properties. In particular, variations in nanoparticle geometry have been shown to modulate protein orientation and secondary structure, even for identical surface functional groups.^32–34^ More recent studies have further highlighted how the shape of a nanoparticle induces distinct conformational responses compared to spherical systems, emphasizing the role of local curvature and contact area at the nanobiointerface.^35^

The aim of this work is to investigate protein corona formation on a model system consisting of the dysopsonin BSA protein and mesoporous silica nanoparticles with an MCM-41-type structure. To investigate the interactions involved in the formation of the protein corona, we compared BSA adsorption on bare (MSN) and amino-functionalized (MSN-NH_2_) nanocarriers at physiological pH 7.5. At this pH, surface functionalization results in a change in the surface charge from negative to positive values,^25^ thereby affecting interactions with BSA and, consequently, the formation of the protein corona for the two materials, which modulates protein adsorption and conformation. First, the physicochemical characterization of MSN and MSN-NH_2_ was carried out by means of transmission electron microscopy (TEM), small-angle X-ray scattering (SAXS), thermogravimetric analysis (TGA), dynamic light scattering (DLS), and zeta potential, to thoroughly investigate the structure and functionalization of the nanoparticles and their colloidal stability. BSA adsorption on MSNs was investigated by zeta potential titrations, quartz crystal microbalance with dissipation monitoring (QCM-D), and circular dichroism (CD) spectroscopy to correlate interfacial forces with conformational changes in BSA upon binding.

## Materials and Methods

### Chemicals

Hexadecyltrimethylammonium bromide (CTAB, >99%), tetraethoxysilane (TEOS, 98%), toluene (C_7_H_8_) (99.8%), 3-aminopropyltriethoxysilane (APTES, >98%), Tris buffer (98%), sodium hydroxide (NaOH), hydrochloric acid (HCl) (37%), and bovine serum albumin (BSA, >98%) were all purchased from Merck (Italy). Absolute ethanol (99.8%) was purchased from Honeywell, and ammonium nitrate (NH_4_NO_3_, 99%) was purchased from CARLO ERBA.

### MSN Synthesis and Functionalization

MSNs were synthesized following a published procedure.^23^ Aminopropyl mesoporous silica nanoparticles (MSN-NH_2_) were obtained using 3-aminopropyltriethoxysilane (APTES) as the functionalizing agent. A mass of 0.5 g of MSN was dispersed in 20 mL of dry toluene in a 50 mL round-bottom flask with 2 necks. The dispersion was stirred at 110 °C under reflux for 12 h. Then, 2 mL of APTES was added to the dispersion and stirred at 280 rpm and 110 °C overnight. The functionalized nanoparticles were then filtered, washed with water and with ethanol, and finally dried under vacuum.

### MSN Characterization

MSN characterization prior to protein adsorption was performed using transmission electron microscopy (TEM, JEOL JEM 2010) at two magnifications. Small-angle X-ray scattering (SAXS) measurements were carried out using an S3-MICRO SWAXS camera system (HECUS X-ray Systems) equipped with a GeniX Cu Kα source (λ = 1.542 Å) operated at 30 kV and 0.4 mA. Samples were measured at room temperature in 2 mm glass capillaries. Thermogravimetric analysis (TGA) of MSN and MSN-NH_2_ was performed under oxygen flow (40 mL min^–1^) from 25 to 850 °C at a heating rate of 10 °C min^–1^ using a PerkinElmer TGA/DSC instrument. DLS measurements were carried out using a Nano ZS analyzer (Malvern) at a nanoparticle concentration of 0.1 mg/mL, dispersed in Milli-Q water, and sonicated for 20 min prior to measurement.

### QCM-D Measurements

The QCM-D measurements were carried out using a Q-Sense E1 (Biolin Scientific) equipped with a peristaltic pump at a flow rate of 0.5 mL/min at *T* = 25 °C. Gold sensors were cleaned with a piranha solution and then thoroughly rinsed with water. BSA and MSN or MSN-NH_2_ were dispersed in Tris buffer at pH 7.5. The following steps were followed to secure protein and nanoparticle adsorption. The procedure used for QCM measurements was designed as follows to monitor the interactions between the MSN and BSA on a gold sensor surface. Stabilization with Tris (0–10 min): 10 mM Tris solution at pH 7.5 was used to achieve baseline signal. Introduction of MSN (10–55 min): The MSN dispersion was flowed to allow adsorption on the Au sensor surface. This phase enables observation of the mass increase associated with the adsorption of the MSN, providing insight into its interaction with the surface. Rinsing with Tris (55–100 min): A Tris rinse was performed to wash away any unbound MSN particles, ensuring that only firmly attached particles remain. This step verifies the stability of the MSN attachment to the surface. Introduction of BSA (100–145 min): The BSA solution was flowed to observe its adsorption to the surface already coated with MSN. The increase in mass indicates the establishment of attractive interactions between BSA and the MSN layer, reflecting how BSA adsorbs onto the modified surface. Rinsing with Tris (145–190 min): A second rinse with Tris was performed to remove any loosely bound BSA molecules, enabling the assessment of the stability of the BSA-MSN interaction. Final rinse with water (190–215 min): A final rinse with water was performed to ensure the removal of any remaining unbound material, leaving only those firmly attached to the surface.

### Zeta Potential Titrations

Zeta potential titrations were carried out by using a Nano ZS analyzer (Malvern). To prepare the MSN-BSA and MSN-NH_2_-BSA complex dispersions, MSN (MSN-NH_2_) and BSA solutions were first prepared separately at 1000 μg/mL for the nanoparticles and 1000, 2000, and 3000 μg/mL for the BSA solutions. The MSN solution was sonicated for 20 min to ensure optimal dispersion of the nanoparticles. Subsequently, 1 mL of MSN and 1 mL of BSA solutions at the initial concentrations were mixed using a magnetic stirrer for 1 h to achieve the final concentrations of 500, 1000, and 1500 μg/mL for BSA and 500 μg/mL for MSN or MSN-NH_2_. The resulting MSN-BSA complex solution was then subjected to zeta potential titration. The solution was brought to pH 3 by adding 2 mL of 0.1 M HCl. Then, various aliquots of 0.1 mM NaOH were added, and the resulting dispersion was measured for the zeta potential, up to a pH of 10.

### Circular Dichroism (CD) Measurements

CD was used to monitor the BSA secondary structure in the presence of MSN/MSN-NH_2_ after 1 h of mixing in a 10 mM Tris buffer at pH 7.5 and after 24 h of mixing with a 10 mM NaCl solution at pH 7.5, using a Jasco-1500 spectrometer with a 10 mm quartz cuvette. Solvent spectra were recorded by using 10 mM Tris buffer or 10 mM NaCl at pH 7.5. The spectra were measured in the wavelength range of 185–300 nm with a resolution of 1 nm and a scanning rate of 50 nm min^–1^. Due to the influence of the solvent, Tris or NaCl, deconvolution was performed in the λ range of 200–250 nm. CD spectra were recorded after subtracting the corresponding solvent baseline, and sample absorbance was kept within the recommended limits for far-UV CD. The mesoporous silica formed stable submicrometric dispersions at the concentrations used (up to 0.5 mg/mL), minimizing potential light scattering effects. Spectra were routinely checked for scattering artifacts (noise increase or baseline distortion), and none were observed. Control measurements of silica dispersions without protein confirmed the absence of CD active signals. To analyze the circular dichroism spectra, CD (mdeg) and wavelength (nm) data were inserted in BeStSel software (available at https://bestsel.elte.hu/index.php). First, BSA (100 μg/mL) was prepared in 10 mM Tris buffer at pH 7.5 and mixed with MSN/MSN-NH_2_ at concentrations of 100 and 200 μg/mL to obtain final nanoparticle dispersion concentrations of 50 and 100 μg/mL. MSN/BSA complexes in NaCl (10 mM) were prepared to ensure a wide range of MSN concentrations in contact with the protein. Specifically, a BSA solution of 100 μg/mL was prepared in 10 mM NaCl to achieve a final concentration of 50 μg/mL after dilution. MSN dispersions of 1500, 1000, and 500 μg/mL were also prepared in 10 mM NaCl to achieve final concentrations of 750, 500, and 250 μg/mL. The final complexes reached a volume of 2 mL.

### Theoretical Modeling of the pH-Dependent MSN Surface Charge

The MSN surface charge was modeled with a pH-dependent charge regulation model,^36,37^ with parameters fitted against experimental zeta potentials. The surface of bare MSN itself is treated as a zwitterionic surface of acidic and basic silanol groups with surface densities of *N*_O,a_ and *N*_O,b_:

$$—\mathrm{SiOH}⇌—{\mathrm{SiO}}^{-}+H^{+,}pK_{O,a}$$ 1a

$$—{\mathrm{SiOH}}^{+}⇌—\mathrm{SiO}+H^{+},pK_{O,b}$$ 1b

The term "–SiO" is not intended to depict a chemical structure. Rather, it is used as a shorthand to indicate a basic surface site in contrast to acidic or amphoteric sites.

In MSN-NH_2_, the surface densities of silanol sites are reduced, replaced by positive amine groups with average surface density *N*_N_:

$$—{{\mathrm{RNH}}_{3}}^{+}⇌—{\mathrm{RNH}}_{2}+H^{+,}pK_{N}$$ 2

The profile of the electrostatic potential ψ­(*x*) close to the surface of the MSN and MSN-NH_2_ particles in 10 mM Tris buffer solution was calculated using a Poisson–Boltzmann model of the electrolyte that explicitly includes H^+^ and OH^–^ ions and buffer species, with bulk ion concentrations set according to the given pH. The surface charge was determined by the charge-regulated equilibria above, eqs 1a, 1b, and 2. The model was solved using an in-house Python library developed for numerically solving the modified Poisson–Boltzmann equation using the finite-element method implemented in FEniCS.^38^ The model allows Mahanty–Ninham ionic dispersion forces^38,39^ to be included, which we compared against the classical purely electrostatic model. The theoretical zeta potentials were determined as the value of the electrostatic potential at the slipping plane,^38^ taken at a distance of the diameter of approximately 3 water molecules (7 Å) from the surface. The charge regulation parameters (pK_*_ and surface densities *N*_O*_ and *N*_N_) were determined by a nonlinear least-squares best fit between theoretical and measured zeta potentials. Fitted charge regulation parameters for bare MSN and MSN-NH_2_ are reported in Table S1 (Supporting Information file). A charge regulation model of the more complex surface charge of BSA was modeled previously.^40,41^

## Results and Discussion

### Physicochemical Characterization of MSN and MSN-NH_2_

MSN and MSN-NH_2_ samples were characterized by TEM, SAXS, and TGA. Figure 1 shows TEM images of MSN (Figure 1A) and MSN-NH_2_ (Figure 1B) at different magnifications with an average diameter of 150 nm and the typical silica nanoparticle internal pore structure. SAXS patterns of MSN and MSN-NH_2_, shown in Figure 1C, report peaks having different intensities associated with the reflections of the planes 100, 110, 200, and 210 typical of a hexagonal arrangement of pores. The distribution of particle size (*d*_H_), obtained by DLS measurements, had a maximum at 145 ± 15 nm for MSN, which increased to 155 ± 30 nm for MSN-NH_2_ due to the grafting of propyl-NH_2_ groups. The amount of the propyl-NH_2_ moiety grafted on MSNs, quantified by thermogravimetric analysis, was 0.646 mmol/g (Figure 1D and Table 1). TGA measurements of MSN and MSN-NH_2_ show a mass loss in the temperature range of 25–110 °C, attributed to the removal of water and to condensation of surface silanol groups. For MSN-NH_2_, an additional mass loss of 6.9% is observed in the temperature range of 110–280 °C, which is attributed to the thermal decomposition of grafted aminopropyl groups. The amount of the propyl-NH_2_ moiety grafted on MSN was quantified from this mass loss and is reported in Table 1.

**Figure 1:**
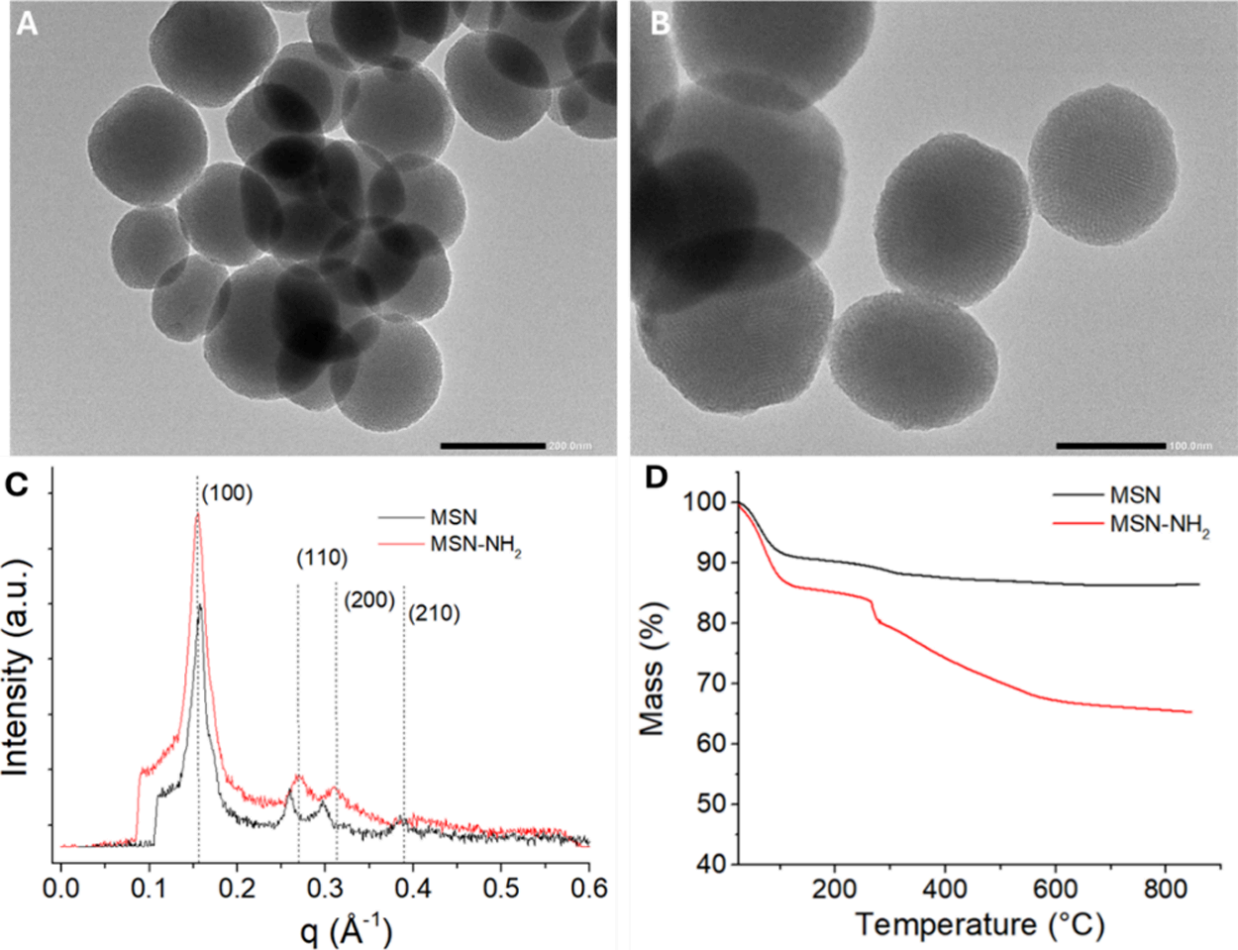
TEM images of MSN at different magnifications (A, B), SAXS measurements (C), and TGA analysis of MSN and MSN-NH_2_ (D).

**Table 1:** Characterization of MSN and MSN-NH_2_: The Lattice Parameter (*a*), Hydrodynamic Diameter (Number Distribution, *d*_H_), Amount of Grafted Propyl-NH_2_ Groups on MSNs, Zeta Potential (ζ), and Isoelectric Point

sample	*a* (nm)	d_H_ (nm)	amount of propyl-NH_2_ (mmol/g)	ζ^a^ (mV)	ζ^b^ (mV)	IEP
MSN	4.7	145 ± 15	-	–21 ± 3	–14 ± 1	4.9
MSN-NH_2_	4.5	155 ± 30	0.646	+22 ± 2	+10 ± 2	7.9

a Zeta potential in water.

b Zeta potential in Tris buffer at pH 7.5.

The zeta potential of MSNs dispersed in Milli-Q water was −21 ± 3 mV due to the dissociation of surface Si–OH groups. After functionalization, the zeta potential of the obtained MSN-NH_2_ sample was +22 ± 2 mV due to the basic behavior of surface amino groups. In 10 mM Tris (pH 7.5), the dispersions remained colloidally stable despite smaller |ζ| values, compared to water. The low ionic strength affords a longer Debye length and effective double-layer repulsion, and the hydrophilic silica surface further disfavors irreversible aggregation; no precipitation or size growth was observed over the experimental time scale.

### Zeta Potential Titrations of MSN, MSN-NH_2_, and Their Complexes with BSA

Zeta potential titrations were performed for bare (MSN), functionalized (MSN-NH_2_) nanoparticles for BSA, and for the protein-nanoparticles complexes obtained by incubating BSA with nanoparticles for 1 h at different BSA:MSN concentration ratios and in the presence of 10 mM TrisH^+^ and Cl^–^ ions. These ions were added to the system to regulate the pH in the subsequent QCM-D and CD experiments. The red curve in Figure 2A shows the zeta potential dependence on pH of bare MSNs. MSNs present a slightly positive zeta potential at acidic pH up to the isoelectric point (IEP ≈ 4.9), after which the nanoparticles assume a negative value of ζ = −14 mV at pH 7.5. The titration curve of MSN-NH_2_ in Figure 2B shows a clear shift of the isoelectric point (IEP ≈ 7.9) due to surface functionalization. The positive zeta potential observed at acidic pH is due to the presence of protonated amino groups on the nanoparticles’ surface. MSN-NH_2_ at pH 7.5 still shows a positive net charge with a zeta potential of +10 mV. At a basic pH of 10, the MSN-NH_2_ sample achieves a negative zeta potential of −26 mV, confirming the presence of residual surface Si–OH groups that have not been functionalized. Zeta potential titration of BSA dissolved in 10 mM Tris-HCl aqueous solution resulted in an IEP ≈ 5.3, slightly higher than what was previously found for BSA in pure water (IEP ≈ 4.7–4.9), as expected due to the adsorption of TrisH^+^ ions on BSA surface.^40^ The divergence between the zeta potential titration curves of BSA and those of the MSN-BSA and MSN-NH_2_-BSA mixtures near and above the nanoparticle isoelectric point can be attributed to incomplete masking of the nanoparticle surface upon protein adsorption. At alkaline pH, BSA adsorbs onto MSN, but the protein layer does not fully shield the deprotonated silanol groups, which remain exposed near the electrokinetic slip plane and contribute to a more negative zeta potential than either BSA or MSN alone. A similar effect is observed for MSN-NH_2_, where deprotonated residual silanol groups are not completely screened by the adsorbed protein. Control titrations of BSA alone were fully reproducible, confirming that the deviations arise from nanoparticle surface chemistry rather than the variability in the protein response.

**Figure 2:**
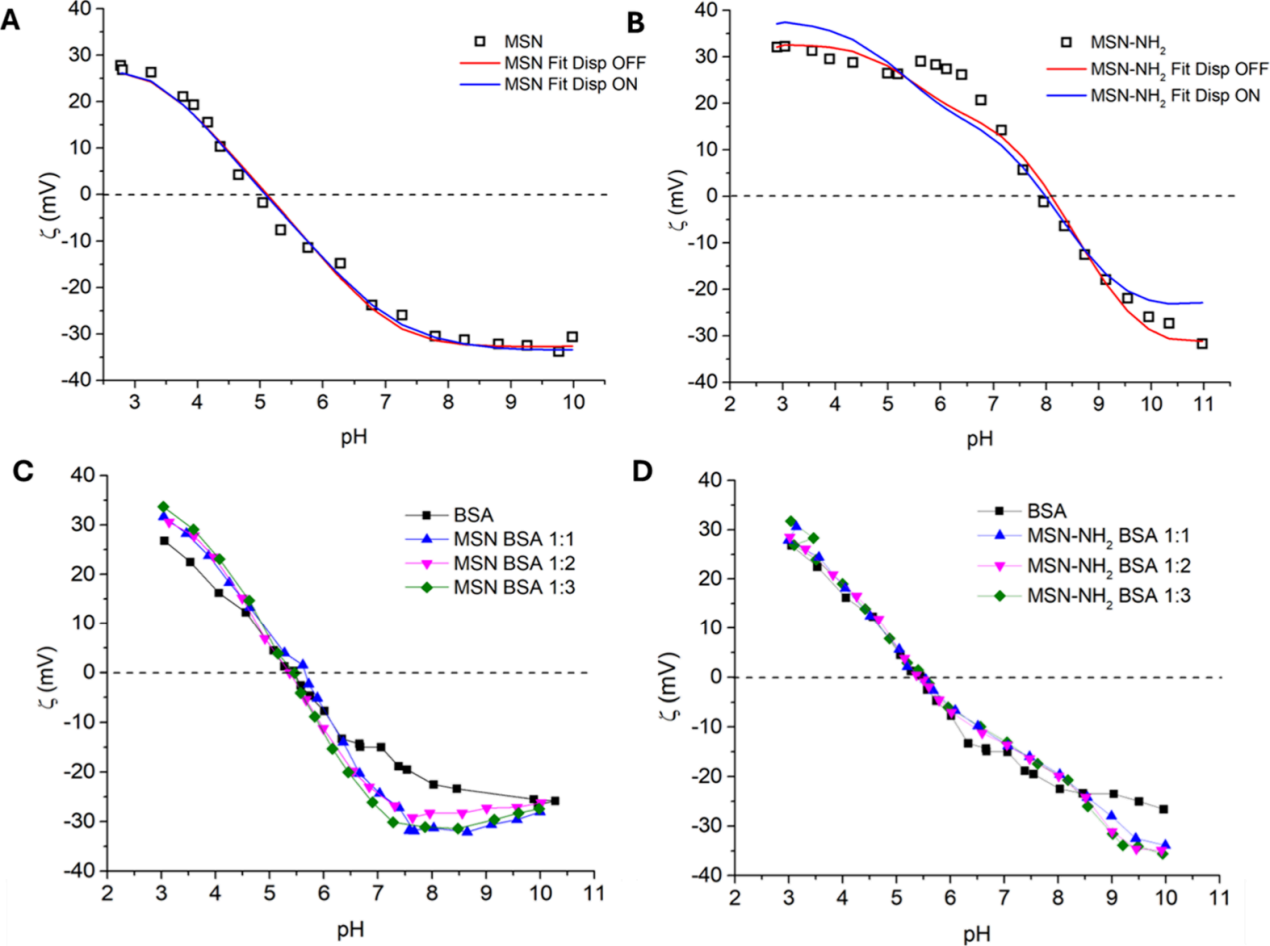
Zeta potential titrations of 500 μg/mL MSN (A) and MSN-NH_2_ (B) in a comparison between experimental and simulated zeta potentials with and without the contribution of dispersion forces. Zeta potential titrations of (C) MSN (500 μg/mL) and (D) MSN-NH_2_ (500 μg/mL) and the respective complexes with different BSA concentrations of 500 μg/mL (1:1), 1000 μg/mL (1:2), and 1500 μg/mL (1:3) in 10 mM Tris-HCl.

p*K*_a_ acid dissociation constants of MSN-NH_2_ were determined through a pH-dependent charge regulation model of the surface charge fitted against experimental zeta potentials. The comparison between the experimental zeta potentials and the theoretical values is shown in Figure 2. For bare MSN, simulations in both cases are close to the experimental values, with no apparent differences between them (Figure 2A). On the other hand, including ionic dispersion forces in the model for the MSN-NH_2_ yields values that are closer to the experimental data (Figure 2B). Activating dispersion forces does not significantly affect the p*K*_a_ values of the groups; its main effect is on the site densities (Table S1). From the fitted parameters, it is evident that the site density of basic −SiO groups decreases from MSN to MSN-NH_2_. This is consistent with the fact that part of the −SiO groups are replaced by −NH_2_ groups, making our model physically robust. Basic −SiO groups are preferentially substituted relative to acidic −SiOH groups, decreasing by an order of magnitude in the classical model and becoming negligible when dispersion forces are included. In contrast, acidic −SiOH groups are only slightly reduced, while the higher density of −NH_2_ sites suggests additional binding to nonactive silanol groups on the MSN surface.

The black curve in Figure 2C,D shows the zeta potential titration of BSA, whereas the other (blue, pink and green) curves show the zeta potential titrations of samples obtained after mixing BSA solutions with nanoparticle dispersions with different BSA/MSN (Figure 2C) and BSA/MSN-NH_2_ (Figure 2D) concentration ratios, namely, 1:1, 1:2, and 1:3 (Tables S2 and S3). In all cases, the titration curves of the BSA–nanoparticle complexes are similar to those of pure BSA, regardless of the considered concentration ratio. We interpret this result as due to the formation of a BSA protein corona on both MSN and MSN-NH_2_ at all concentration ratios, with the consequent modification of the chemical and electrical features of the nanoparticles’ surface. The similarity between the BSA/MSN and BSA/MSN-NH_2_ curves and that of free BSA is a sign of the occurrence of a surface protein layer on both bare and functionalized MSNs. Indeed, the IEP of MSN-NH_2_ decreases from ≈7.9 to ≈5.3 after BSA adsorption as a result of the occurrence of attractive interactions among them. More interestingly, BSA is adsorbed to a similar extent on bare MSNs, which is an unexpected result if only surface potentials are considered. A detailed discussion of the interaction mechanisms involved is provided in subsequent sections.

### BSA Adsorption on MSN and MSN-NH_2_ through QCM-D Measurements

The adsorption of BSA on bare and functionalized MSNs in 10 mM Tris buffer at pH 7.5 was then investigated with QCM-D (Scheme 1). QCM-D measurements enable the quantification of the adsorbed amounts of MSN and MSN-NH_2_ on the gold sensor as well as that of the BSA protein onto the first nanoparticle layer. Figure 3 shows the comparison, in terms of the frequency difference Δ*f* and the corresponding adsorbed mass as a function of time, for BSA interaction with MSN (Figure 3A,C) and MSN-NH_2_ (Figure 3B,D). The adsorbed amount (ng/cm^2^) was calculated through the Sauerbrey equation^42^ as detailed in the Supporting Information.^43^

**Figure 3:**
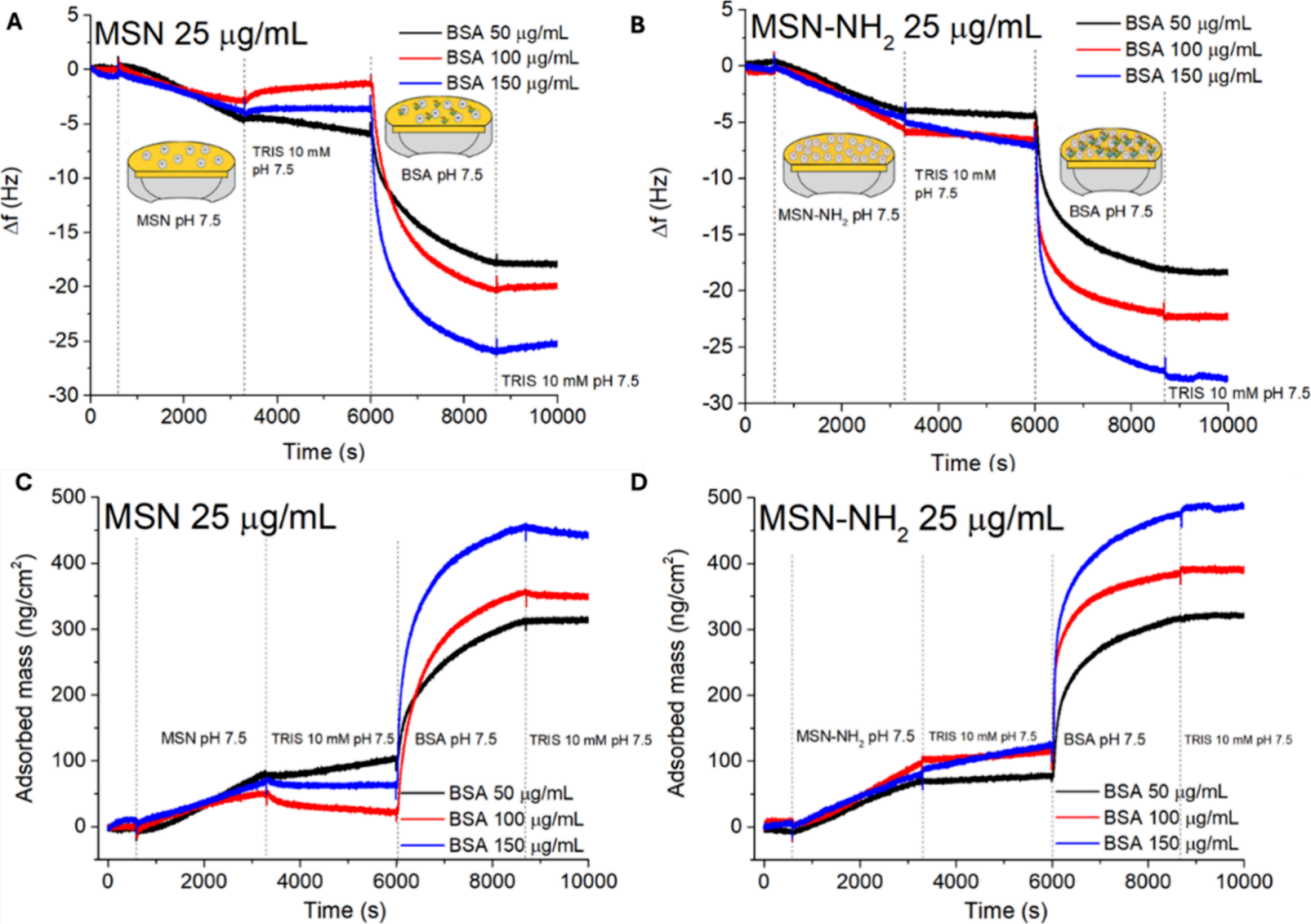
QCM-D measurements of changes in frequency (Δ*f*) vs time for MSN (A) and MSN-NH_2_ (B) at 25 μg/mL with different concentrations of BSA. The corresponding BSA adsorbed mass (ng/cm^2^) is shown in (C) for MSN and in (D) for MSN-NH_2_. The first 600s were used to rinse the sensors with Tris at 10 mM pH 7.5.

**Scheme 1:**
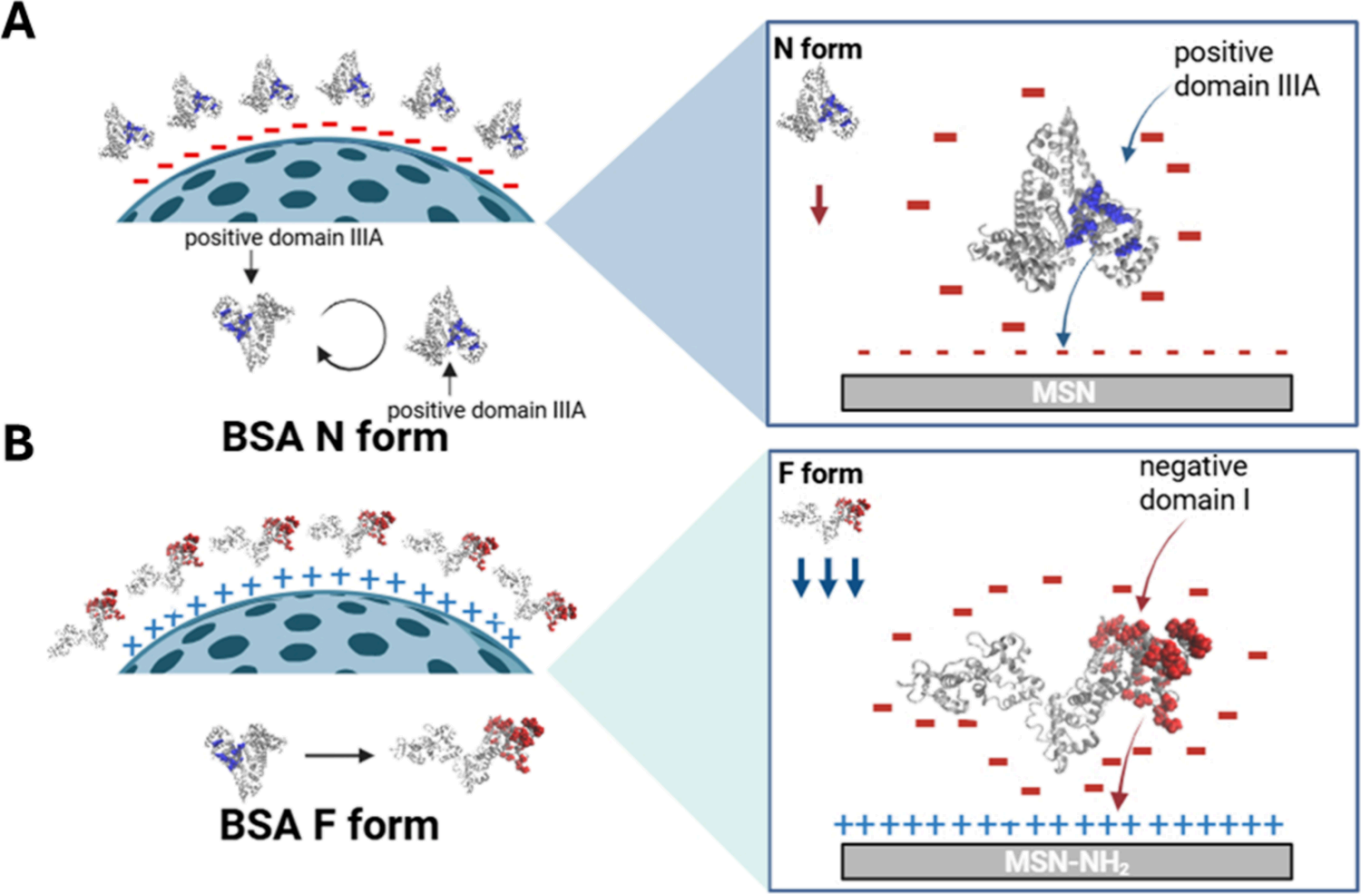
Expected Interactions between BSA Molecules and Bare MSN (A) or MSN-NH_2_ (B) at pH 7.5^a^

a Under these conditions, BSA is repelled by bare MSN and attracted to the MSN-NH_2_ surface. The N (native) form of BSA was taken from PDB entry 3V03 (chain A, monomeric), whereas the F (fast) form was reconstructed based on the structure reported by Baler et al.^16^ Positively charged (blue) and negatively charged (red) residues are highlighted using VMD.

Measurements were carried out by keeping constant the nanoparticle (either MSN or MSN-NH_2_) concentration at 25 μg/mL while three different concentrations of BSA, namely, 50, 100, and 150 μg/mL, were used (Table S4). The QCM-D gold sensor was initially rinsed with 10 mM Tris buffer at pH 7.5, and then, the nanoparticle dispersion was flowed from 600 to 3000 s, resulting in a frequency decrease due to the adsorption of either MSN (Figure 3A) or MSN-NH_2_ (Figure 3B) on the gold sensor. The adsorbed amounts of nanoparticles on the gold sensor and of BSA on the nanoparticle layer are reported in Table 2.

**Table 2:** QCM Adsorption Data in 10 mM Tris Buffer at pH 7.5 and Adsorbed Amount of MSN and MSN-NH_2_ (25 μg/mL) on the Gold Sensor followed by the Adsorption of BSA Protein on the Nanoparticle Layer

sample	concentration (μg/mL)	Δm (ng/cm^2^)	time (s)
MSN on gold	25	69 ± 14	600–3300
MSN-NH_2_ on gold	25	87 ± 12	600–3300
BSA on MSN	50	228 ± 9	6000–8800
BSA on MSN-NH_2_	50	246 ± 4	6000–8800
BSA on MSN	100	324 ± 7	6000–8800
BSA on MSN-NH_2_	100	286 ± 5	6000–8800
BSA on MSN	150	392 ± 6	6000–8800
BSA on MSN-NH_2_	150	380 ± 12	6000–8800

After 3300 s, a rinse with 10 mM Tris buffer at pH 7.5 was performed to remove the unbound nanoparticles. At 6000 s, BSA solutions, at the three different concentrations in 10 mM Tris buffer at pH 7.5, were flowed onto the adsorbed layer of nanoparticles (Figure 3A,B). After 8800 s, a final rinse with Tris buffer was performed to remove unbound BSA molecules. However, no significant differences in mass for adsorbed BSA were observed on either MSN (Figure 3C) or MSN-NH_2_ (Figure 3D), confirming a strong interaction between BSA and the underlying layer of adsorbed nanoparticles. On bare gold, BSA adsorbs with Δ*m* ≈ 100–110 ng/cm^2^ (Figure S1), consistent with a monolayer of protein on the sensor, whereas higher, nanoparticle-dependent Δ*m* values on MSN/MSN-NH_2_-coated sensors indicate BSA adsorption onto the nanoparticles layer.

Adsorbed amount data, listed in Table 2, show that with bare MSN 25 μg/mL and the increase in BSA concentration from 50 to 100 μg/mL resulted in an increase in the adsorbed amount for BSA (228 ± 9 and 324 ± 7 ng/cm^2^, respectively), and a slight increase in mass (392 ± 6 ng/cm^2^) was obtained for BSA (150 μg/mL) (Figure 3A). For MSN-NH_2_ 25 μg/mL, an increasing adsorbed amount of BSA was observed when protein concentration was flowed at 50, 100, and 150 μg/mL. In particular, the adsorbed amount of BSA at 50 μg/mL on MSN-NH_2_ was 246 ± 4 ng/cm^2^ and increased to 286 ± 5 ng/cm^2^ and 380 ± 12 ng/cm^2^ for BSA concentrations of 100 and 150 μg/mL, respectively. Results in Table 2 describe, from a quantitative point of view, what was observed only qualitatively for the zeta potential titrations (Figure 2). That is, BSA is adsorbed both on MSN and MSN-NH_2_ samples, although with a preference for the latter (Scheme 1). This result is explainable due to an attractive electrostatic interaction between negatively charged BSA and positively charged MSN-NH_2_ at pH 7.5.^18^ However, the relatively high amount of BSA adsorbed on bare nanoparticles is rather unexpected since, unlike MSN-NH_2_, both MSN and BSA molecules are negatively charged at pH 7.5, and a net repulsive electric-double-layer interaction would be expected.

Figure 4 shows the effect of the nanoparticle concentration on BSA adsorption. Adsorbed mass of nanoparticles depends also on their type, with a preferred adsorption on a gold sensor for the functionalized silica nanoparticles (Figure 4 A,B). Adsorbed mass of BSA changes slightly regardless of the flowed concentration of MSN but it increases with MSN-NH_2_ concentration ranging from 24628 ± 4 ng/cm^2^ for MSN-NH_2_ 25 μg/mL up to 481 ± 8 ng/cm^2^ of adsorbed protein for MSN-NH_2_ 150 μg/mL. Unlike MSN, when the concentration of MSN-NH_2_ increases, the amount of BSA adsorbed increases significantly, likely due to the formation of soft, highly hydrated protein layers (Figure 4F).^44–46^ Bare MSNs did not present significant differences in their adsorbed amount. At the same time, MSN-NH_2_ adsorption increased with increasing their concentration, suggesting a stronger interaction between the gold surface and the amino-functionalized nanoparticles (Tables S5 and S6).^47^ The dissipation (Δ*D*) response (Figure 4E,F) provides insight into the viscoelastic nature of the adsorbed layer by reflecting the energy lost per oscillation cycle. The adsorbed mass measured on the QCM-D sensor includes a certain number of water molecules. More rigid and less hydrated films typically exhibit low dissipation, whereas highly hydrated layers result in higher dissipation due to greater viscoelastic energy losses. Consequently, low dissipation values correspond to the formation of rigid layers, whereas high dissipation values are associated with the formation of flexible, hydrated films. For MSN (Figure 4E), Δ*D* values remained low (Δ*D* < 3 × 10^–6^). Nearly independent of NP concentration, indicating the formation of a thin, rigid, and compact protein layer with minimal water entrapment.^10,47^ Conversely, for MSN-NH_2_ (Figure 4F), Δ*D* markedly increased with nanoparticle concentration (up to Δ*D* 20 × 10^–6^), corresponding to the formation of a soft, hydrated, and dissipative film.

**Figure 4:**
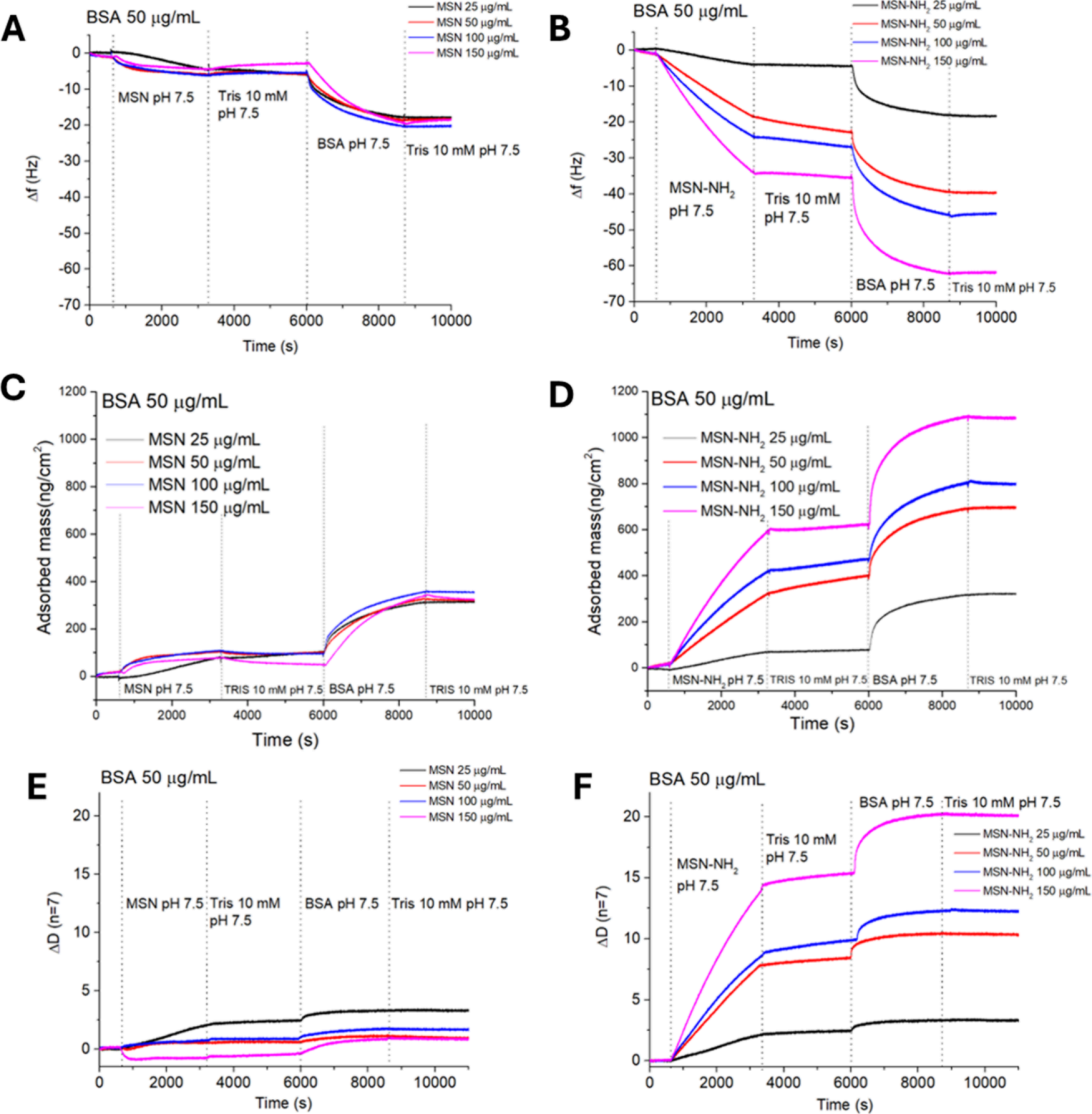
QCM-D measurements of changes in frequency (Δ*f*) vs time for MSN (A) and MSN-NH_2_ (B) at different nanoparticle (NPs) concentrations with BSA (50 μg/mL) in 10 mM Tris buffer at pH 7.5. QCM-D measurements of changes in adsorbed mass (Δ*m*) for MSN (C) and MSN-NH_2_ (D) at different nanoparticle (NP) concentrations with BSA of 50 μg/mL in Tris buffer 10 mM at pH 7.5. QCM-D measurements of changes in dissipation (Δ*D*) versus time for (E) MSN and (F) MSN-NH_2_ at different nanoparticle concentrations. Measurements were performed in 10 mM Tris buffer (pH 7.5) with BSA at 50 μg/mL.

### BSA Conformation Changes due to MSN and MSN-NH_2_

CD analysis provides valuable insights into protein–nanoparticle interactions, revealing how surface properties and nanoparticle functionalization influence the BSA conformation. To analyze the effect of MSN and MSN-NH_2_ on the secondary structure and conformation of BSA, CD measurements were performed depending on the nanoparticle-to-BSA ratio. Changes in characteristic bands are associated with rearrangements of the protein’s secondary structure; in particular, a decrease in the negative peak at 208 nm indicates a reduction in α-helix content, suggesting peptide strand rearrangement.

Figure 5 shows the CD spectrum for the nanoparticle system at 50 and 100 μg/mL BSA concentrations in Tris solution. The effect of nanoparticle interaction is particularly visible at lower protein levels. In the case of BSA, the secondary structure is primarily dominated by the α-helix (60%), with a significantly smaller contribution from the β-sheet (less than 10%).^48^ The CD-UV spectrum of native BSA in solution exhibits a typical profile characterized by one positive component at 197 nm and two negative components at 208 (resulting from the π→π* transition) and 222 nm (resulting from the n→π* transition). After protein adsorption on the nanoparticle surface, the total intensity of these signals decreased and their relative intensity also changed. Due to the influence of the solvent (Tris or NaCl solutions), deconvolution was performed in the λ range of 200–250 nm.

**Figure 5:**
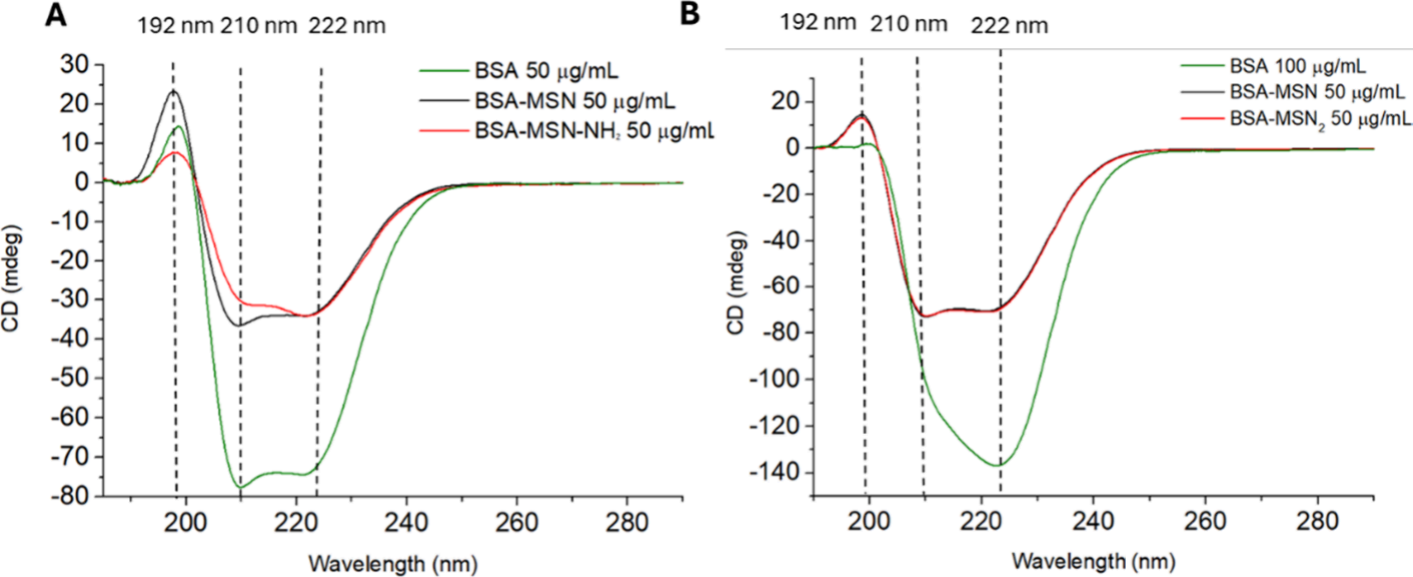
CD spectra of BSA at 50 μg/mL (A) and 100 μg/mL (B) after 1 h of adsorption on MSN and MSN-NH_2_ (50 μg/mL) in 10 mM Tris buffer at pH 7.5.

Figure 6 shows the well-organized secondary structures of BSA after adsorption on the surfaces of both types of molecules. With increasing MSN concentration, there is a general change in the CD signal intensity, evident in the negative band around 222 nm, which reflects the α-helix content in the BSA structure.^33^ Under these conditions, all BSA spectra do not show a significant shift in the positions of all peaks. The initial BSA spectrum is consistent with the literature values; however, with an increase in the number of nanoparticles in the system relative to the protein, a change in the relative intensity of the minima at 208 and 222 nm is observed (Figure 5A,B). For the initial structures, the ratio of the intensities of the negative peaks is >1.

**Figure 6:**
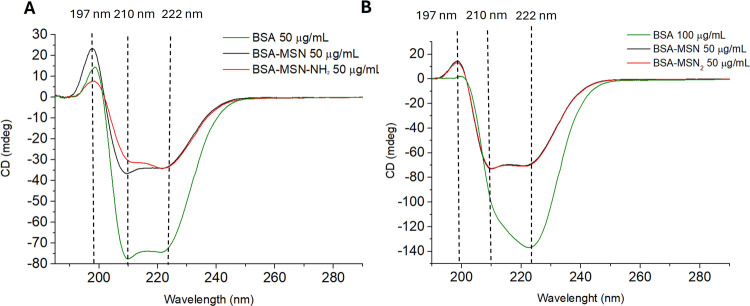
CD spectra of BSA (50 μg/mL) with MSNs (A, B) and MSN-NH_2_ (C, D) at different concentrations (250, 500, and 750 μg/mL) immediately after mixing (A, C) and after 24 h of adsorption at 4 °C (B, D). BSA in 10 mM NaCl, pH 7.5, is shown in green.

This change is evident in the case of BSA interaction with positively charged MSN-NH_2_ nanoparticles (Figure 6C,D). The relative amount of α-helices decreased in favor of β-sheets. The initial α-helix content drops from 60% to 24–26% (Table 3) in the case of the BSA system with 750 μg/mL MSN-NH_2_. As the α-helix content decreases, the β-sheet content increases from a few percent to 22–24%. The CD spectra in Figure 6 suggest the occurrence of different types of interactions between positive (MSN-NH_2_) and negative (MSN) nanoparticles and BSA molecules. CD spectroscopy cannot be used to quantify the fraction of BSA adsorbed onto MSN or MSN-NH_2_ because the absolute CD intensity is strongly affected by the protein orientation, local dielectric environment, and nanoparticle–protein coupling. These interaction-induced effects alter the spectral amplitude independently of secondary structure, making it impossible to separate contributions from free and adsorbed BSA. Therefore, CD analysis is suitable for identifying qualitative structural trends, but not for estimating adsorption efficiency. Quantitative protein uptake was instead determined using QCM-D, which directly reports the amount of BSA bound to the nanoparticles.

**Table 3:** α-Helix and β-Sheet Percentage of the BSA (50 μg/mL) Secondary Structure Obtained by Deconvolution of CD Spectra^a^ after 24 h Adsorption on MSN and MSN-NH_2_ (750 μg/mL) at pH 7.5 in NaCl (10 mM)

sample	α-helix (%)	β-sheet (%)	random coil (%)
free BSA	60	<5	27
BSA-MSN	59–54	<10	21–28
BSA-MSN-NH_2_	24–26	22–24	37–41

a The spectra were deconvoluted using BeStSel server software.

### Mechanistic Insights into Protein Corona Formation

The results above demonstrate that the positive/negative nature of the nanoparticles’ surface charge induces distinct behavior in the adsorbing BSA molecules. The analysis of the zeta potential titration curves provides important insight into the nature of the interactions between BSA and MSN or MSN-NH_2_. Because BSA and bare MSN have similar isoelectric points, they carry the same negative charge over most of the investigated pH range (Figure 2A,C). Under these conditions and in particular at pH 7.5, the pH used for QCM-D and CD measurements, a predominantly repulsive electric double-layer interaction would be expected, and negligible BSA adsorption should occur if electrostatics were the only driving force. In contrast, MSN-NH_2_ has an isoelectric point of around 7.9 and is positively charged at pH 7.5, while BSA remains negatively charged; attractive electrostatic forces therefore explain the higher adsorption observed on MSN-NH_2_. However, our experimental results clearly show that BSA also adsorbs onto bare MSN, albeit to a lesser extent. QCM-D measurements confirm stable BSA binding on MSN across all tested concentrations, indicating that additional nonelectrostatic contributions, such as dipolar orientation effects, charge-patch interactions, and short-range attractive forces, must assist protein adsorption even in the presence of mean field electric double-layer repulsion. The adsorption mechanism is intrinsically pH-dependent because both the charge distribution of BSA and the protonation state of MSN and MSN-NH_2_ vary with pH. At pH 7.5, MSN-NH_2_ promotes adsorption through attractive electrostatics, whereas adsorption on negatively charged MSN is driven by a combination of orientation-dependent electrostatics and short-range interactions. Although a full pH-dependent analysis is beyond the scope of this work, we note that the balance between these interactions is expected to vary with pH. In a recent study, we investigated the adsorption of BSA on MSN-NH_2_ at pH 7.15, using different buffers at varying concentrations.^18^ In that work, the buffers modulated BSA adsorption by screening the surface potential with buffer specificity, so that the adsorption was explained by the simultaneous action of the electric double-layer and van der Waals forces. In our system, the ionic strength is low (10 mM Tris), which increases the range and magnitude of electrostatic interactions due to the longer Debye length. Under these conditions, electrostatics is expected to dominate the interaction profile, while van der Waals forces, although always present and not negligible in an absolute sense, play a comparatively smaller role in determining the overall adsorption behavior.

To explain the experimental results, we must consider the zwitterionic nature of BSA and its asymmetric charge distribution; indeed, at pH 7.5, where BSA has a negative net charge (Scheme 1A), and there are positively charged structural domains that could be involved in an attractive interaction with the negatively charged silica surface.^9,49^ BSA is composed of three domains, each divided into two subdomains.^47^ Jachimska et al. reported that, at pH 7, BSA has a net charge of −17, and each subdomain has a specific net charge. At pH 7.5, each of these domains has a distinct net charge. Domain III (particularly subdomain IIIA) is positive, while domains I and II are negative under these conditions.^9,50^ In the case of negatively charged MSNs, domain III could be responsible for interactions with the nanoparticles. This interaction can be better understood by considering BSA molecules as dipoles oriented in the direction of the electric field generated by the negatively charged MSN nanoparticles. Repulsive and attractive energy terms due to the electric double layer and van der Waals interactions from DLVO theory have previously been implemented to describe protein adsorption on surfaces.^51,52^ However, to the best of our knowledge, an attractive energetic term considering the dipolar nature of proteins and charged surfaces has not yet been implemented.

Norde^53^ classified proteins as “hard” or “soft”. Hard proteins are structurally rigid and adsorb mainly on hydrophobic or hydrophilic surfaces only when electrostatic interactions are favorable. Soft, flexible proteins with lower rigidity can undergo partial unfolding, enabling adsorption even under less electrostatically favorable conditions. BSA is a soft, flexible protein whose conformation is dependent on pH. At pH 7.5, it is in the N (native) isomeric form,^50,54,55^ whereas at pH 4, it turns to the F (fast) and at around pH <4 to the E (elongated) conformation.^56^ The F and E forms differ from the N form for both the hydrodynamic size and exposure of surface domains (Figure 7B). The BSA secondary structure in its possible conformational forms (Figure 7A,B) has been studied through CD spectroscopy (Table 4).^57^

**Figure 7:**
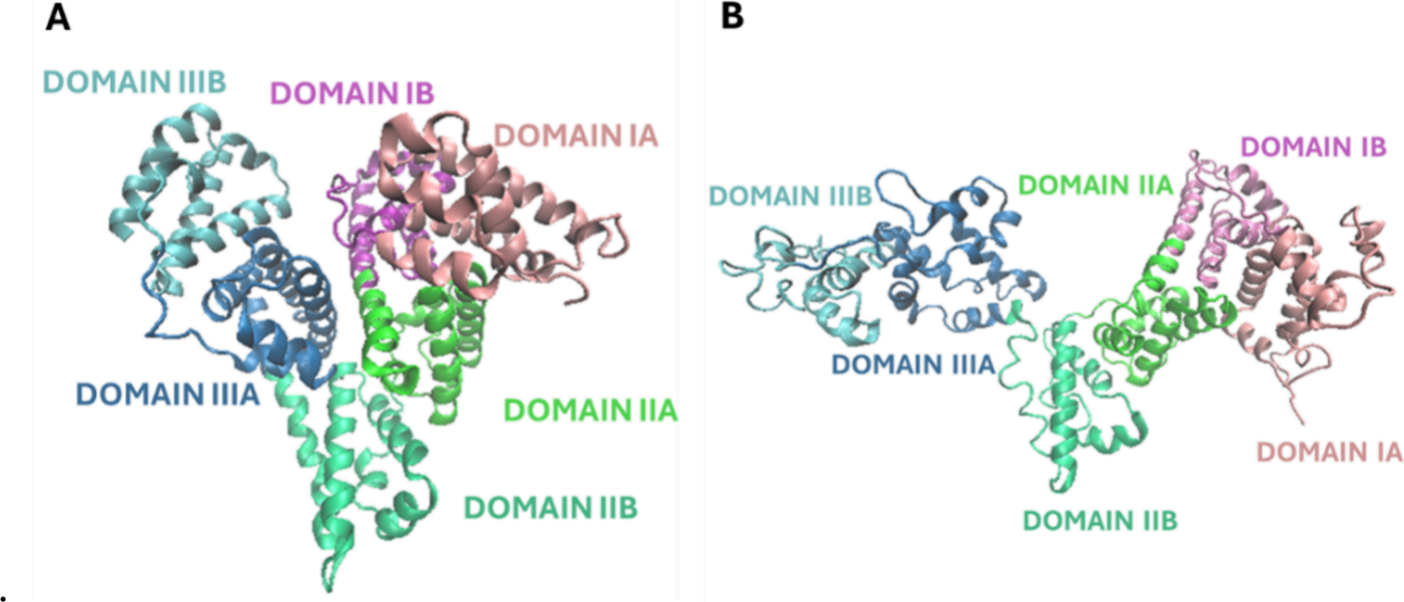
Schematic representation of BSA in its native (N) form (A) and fast (F) form (B), generated by using Visual Molecular Dynamics (VMD). The N form corresponds to the monomeric structure from PDB entry 3V03 (chain A), whereas the F form was reconstructed based on the model reported by Baler et al.^16^ Protein domains are color-coded as follows: domain I in pink (IA, pink; IB, purple), domain II in green (IIA, light green; IIB, green), and domain III in blue (IIIA, blue; IIIB, light blue).^16,50^

**Table 4:** Secondary Structure of Different BSA Forms Existing at Different pH

form	pH range	α-helix (%)	β-sheet (%)	random coil	ref.
N	4–9	∼60–66	∼20–25	∼15–20	^58^ and^59^
F	2,3–9,10	∼40–45	∼20	∼35	^60^
E	<2 to >11	∼30–40	∼18		^61^

CD spectra suggest (Figure 6) that BSA interacts differently with the two types of nanoparticles, as confirmed by a different degree of distortion of its secondary structure (Scheme 1). In particular, the CD spectrum of the BSA-MSN complex is similar to that of free BSA, suggesting that the protein keeps its N (native) form when interacting with negatively charged MSNs.^58–61^ By contrast, the interaction with the positively charged MSN-NH_2_ results in a substantial distortion of the BSA structure, likely promoting a situation close to that of the F (fast) or E (elongated) forms. In particular, in the case of the MSN-NH_2_ sample, the negatively charged domains of BSA will be more responsible for adsorption onto the surface of these particles. Because domain I, together with domain III, exhibits a high conformational flexibility,^62,63^ this may result in evident changes in the overall protein structure. The significant decrease in the 208/222 nm intensity ratio reflects the formation of β-sheet structures, which is observed for the adsorption of BSA onto positively charged MSN-NH_2_.

This model of different interactions between BSA and MSN and MSN-NH_2_ seems to be confirmed by the dissipation plot. The low Δ*D* values observed for MSN indicate the formation of a rigid, compact, and poorly hydrated protein layer, consistent with the limited structural rearrangements of BSA. In contrast, the substantial increase in Δ*D* for MSN-NH_2_ reveals the formation of a soft, viscoelastic, and highly hydrated film. Moreover, for MSN-NH_2_, Δ*f* and Δ*D* seem to be NP concentration-dependent before BSA addition, indicating enhanced affinity of the functionalized surface for the gold sensor. This behavior reflects a stronger electrostatic attraction between BSA and the positively charged surface, promoting partial unfolding of the protein, in agreement with the CD evidence of β-sheet enrichment. Overall, these results demonstrate that the nanoparticle surface charge not only determines the extent of adsorption but also influences the orientation and conformation of the resulting protein corona. Further efforts are needed to theoretically incorporate an energy term related to the dipole moment of proteins. In contrast to other colloidal objects, proteins have asymmetric heterogeneous charge distributions and can assume different conformations. The adsorption of BSA onto MSN and MSN-NH_2_ at pH 7.5 cannot be explained solely by mean-field electric double-layer interactions. Although both BSA and MSN are negatively charged at this pH, the strong charge anisotropy of BSA and its permanent dipole moment allow positively charged patches (in particular, domain IIIA) to orient favorably toward the negatively charged silica surface, enabling adsorption in agreement with zeta potential and QCM-D results. In parallel, short-range attractive forces including van der Waals and hydration interactions also contribute, becoming more relevant for MSN-NH_2_ where partial unfolding detected by CD exposes additional hydrophobic residues and leads to softer, more dissipative layers. Overall, BSA adsorption likely arises from a cooperative balance of orientation-dependent electrostatics and short-range forces modulated by nanoparticle surface chemistry.

## Conclusions

In this work, we investigated the formation of the BSA-based protein corona on bare and amino-functionalized mesoporous silica nanoparticles (MSN and MSN-NH_2_) by combining TEM, SAXS, TGA, DLS, zeta potential characterization with QCM-D (adsorbed mass and viscoelasticity), and CD spectroscopy (secondary structure). BSA adsorbs on both materials, with higher uptake on MSN-NH_2_, consistent with attractive electrostatics at pH 7.5, and with partial unfolding leading to softer, more hydrated films. On negatively charged MSN, measurable adsorption occurs despite net charge repulsion; this behavior is best rationalized by a cooperative mechanism in which orientation-dependent electrostatics (dipole/charge-patch effects) acts together with short-range attractive forces (e.g., van der Waals and hydration), yielding more compact, less dissipative layers. Overall, the surface charge and chemistry govern not only the extent of adsorption but also the structural and mechanical nature of the corona. We recognize that our data set does not, by itself, isolate the individual energetic contributions. A definitive quantification of the roles played by electrostatics (including dipolar/patch-charge alignment), van der Waals, hydrophobic, and hydration forces will require systematic variations of the pH, ionic strength, and buffer identity. These experiments constitute a clear priority for future work aimed at refining the microscopic description of albumin coronas at charged silica interfaces.

## Supplementary Material



## Notes

Detailed QCM-D analysis of adsorbed mass, charge regulation model parameters (Table S1), zeta potential titration values of BSA after adsorption on MSN (Table S2) and MSN-NH_2_ (Table S3), adsorbed amounts of BSA on MSN and MSN-NH_2_ obtained from QCM-D measurements from the data in Figure 3 (Table S4) and Figure 4 (Table S5), and analysis and calculation of the estimated number of adsorbed BSA molecules per nanoparticle and the corresponding adsorbed amounts of BSA on MSN and MSN-NH_2_ (Table S6) (PDF)
