# Correction to “Mechanistic Insights into Protein Corona Formation: The Surface Charge of Mesoporous Silica Nanoparticles Determines the Orientation and the Conformation of Adsorbed BSA Protein”

**DOI:** 10.1021/acs.langmuir.6c03374

**Published:** 2026-06-17

**Authors:** Alessandra Ballicu, Gaia M. Meloni, Matteo Farci, Davide Tocco, Marco Piludu, Drew F. Parsons, Cristina Carucci, Barbara Jachimska, Andrea Salis

Due to a production error, an incorrect version of Figure 5 was included in the published article, and the corrected graphic for Figure 5 was instead erroneously placed in Figure 6. As a result, the correct graphics for both Figure 5 and Figure 6 were not shown as intended in the final published version.

The correct Figure 5 and Figure 6 together with their captions are shown below.

**Figure 5:**
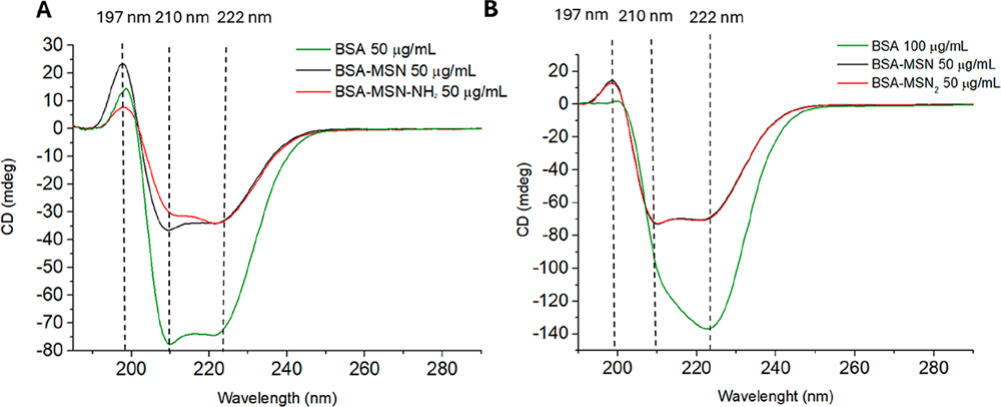
CD spectra of BSA at 50 μg/mL (A) and 100 μg/mL (B) after 1 h adsorption on MSN and MSN-NH_2_ 50 μg/mL in Tris buffer 10 mM at pH 7.5.

**Figure 6:**
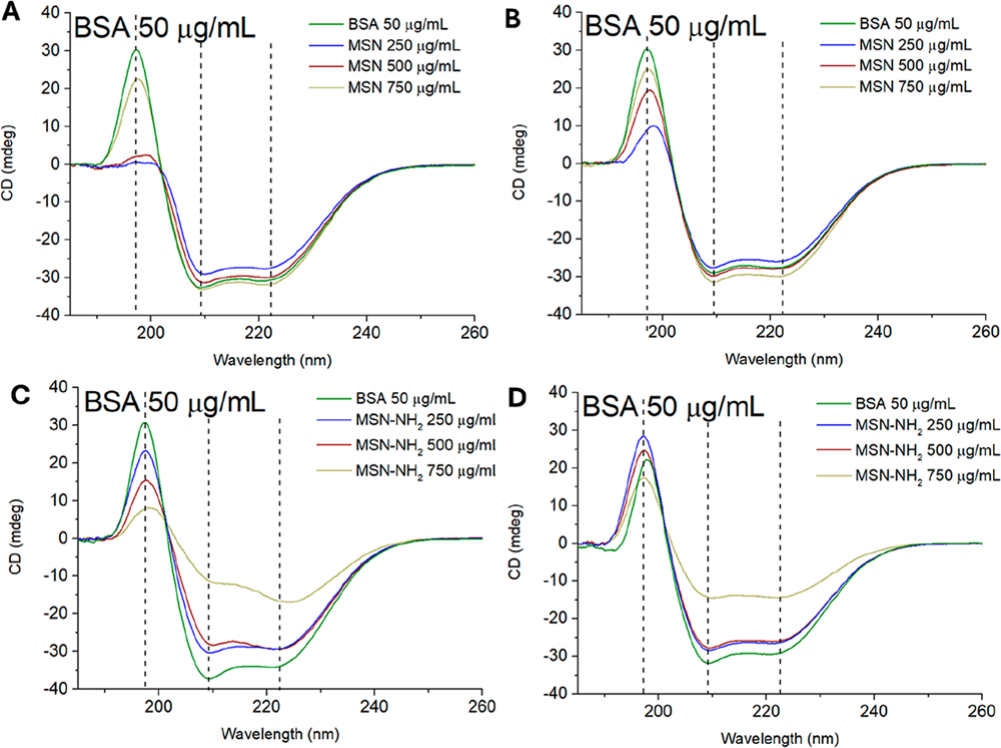
CD spectra of BSA (50 μg/mL) with MSNs (A, B) and MSN-NH_2_ (C, D) at different concentrations (250, 500, and 750 μg/mL) immediately after mixing (A, C) and after 24 h adsorption at 4 °C (B, D). BSA in 10 mM NaCl, pH 7.5, is shown in green.

