# Supplementary material for: Mechanistic Insights into Protein Corona Formation: The Surface Charge of Mesoporous Silica Nanoparticles Determines the Orientation and the Conformation of Adsorbed BSA Protein
Source: Langmuir. 2026 Mar 27;42(13):9170–82. doi: 10.1021/acs.langmuir.5c06171 (PMC13063808; doi:10.1021/acs.langmuir.5c06171)
Supplement: Supplementary file 1 [file la5c06171_si_001.pdf]

Supporting information

Mechanistic Insights into Protein Corona Formation: The Surface Charge of Mesoporous Silica Nanoparticles Determines the Orientation and the Conformation of Adsorbed BSA Protein

Alessandra Ballicu,[a]† Gaia M. Meloni,[a]† Matteo Farci,[a] Davide Tocco,[a] Marco Piludu,[b] Drew F. Parsons,[a] Cristina Carucci[a] Barbara Jachimska,*[c] and Andrea Salis*[a]*

[a] Department of Chemical and Geological Sciences, University of Cagliari & CSGI, Cittadella Universitaria, S.S. 554 bivio Sestu, 09042 Monserrato (CA), Italy.
[b] Department of Biomedical Sciences, University of Cagliari, Cittadella Universitaria, S.S. 554 bivio Sestu, 09042 Monserrato (CA), Italy.
[c] Jerzy Haber Institute of Catalysis and Surface Chemistry Polish Academy of Sciences, Krakow, Poland.

† A.B. and G.M.M. contributed equally to the work.

QCM-D analysis of adsorbed mass

MSN, MSN-NH_2, and BSA adsorption were analyzed using QCM-D. For rigid and thin layers, the Sauerbrey model[1] allows the correlation between the frequency shift and the adsorbed mass through the following equation:

$$\Delta m = -\frac{C}{n}\Delta f \qquad \text{S1}$$

Where Δm is the adsorbed mass per unit area (ng·cm^{-2}), Δf is the frequency shift (Hz), C is the crystal sensitivity constant (17.7 ng·cm^{-2}·Hz^{-1} for 5 MHz quartz crystal), and n is the overtone number (here 7). In the adsorption experiment, the nanoparticles were first injected, followed by a 10 mM Tris buffer at pH 7.5, rinsing, and an additional BSA injection. The following equation was then used to calculate the mass in each measurement:

$$\Delta m_{BSA} = -\frac{C}{n}(f_2 - f_1) \qquad \text{S2}$$

Where f_2 is the frequency (Hz) at 8800 s, corresponding to the end of BSA adsorption step, and f_1 is the frequency (Hz) at 6000 s, corresponding to the beginning of BSA adsorption. The corresponding mass difference can also be expressed as:

$$\Delta m_{BSA} = \mathrm{m}_{postBSA} - \mathrm{m}_{preBSA} \qquad \text{S3}$$

Where $\mathrm{m}_{postBSA}$ and m_{preBSA} are the calculated adsorbed mass per unit area (ng/cm^{-2}) at 8800 s and at 6000 s, respectively. To calculate the second adsorbed layer, the BSA film was considered rigid and firmly attached to the nanoparticles' first layer, thus allowing the use of the Sauerbrey model. In case of a viscoelastic film, the protein mass would be underestimated with a % difference that is proportional to the BSA concentration flowed onto the nanoparticles.[2]

Table S1. Modeling of zeta potential titration curves. Charge regulation model parameters (number of sites, N, and pK_a) with and without ionic dispersion forces.

Sample	Site	Dispersion forces OFF		Dispersion forces ON	
		**N [sites/nm^2]**	**pK_a**	**N [sites/nm^2]**	**pK_a**
MSN	-SiOH	0.07	5.8	0.07	5.9
	-$SiOH_2^+$	0.06	4.5	0.10	4.4
MSN-NH_2	-SiOH	0.04	5.8	0.06	5.9
	-$SiOH_2^+$	0.006	4.5	1E-10	4.4
	-$SiNH_3^+$	0.07	8.2	4.4	8.3

Table S2. Zeta potential titration values of BSA after adsorption on MSN in a range of BSA concentrations.

pH	BSA	pH	MSN	pH	MSN: BSA 1:1	pH	MSN: BSA 1:2	pH	MSN: BSA 1:3
3.05	26.8	2.77	27.8	3.04	31.7	3.14	30.6	3.03	33.7
3.53	22.5	2.8	26.8	3.46	28.3	3.6	27.8	3.59	29.1
4.06	16.2	3.26	26.3	3.87	23.8	3.97	23.5	4.07	23.1
4.56	12.3	3.76	21.1	4.25	18.3	4.49	15.2	4.62	14.7
5.07	4.51	3.94	19.3	4.63	13.3	4.91	6.92	5.15	3.83
5.27	1.26	4.16	15.6	5.27	3.98	5.37	-0.042	5.46	-0.0737
5.44	0.298	4.36	10.3	5.61	1.52	5.67	-5.42	5.57	-4.1
5.57	-2.56	4.65	4.2	5.72	-2.32	5.99	-11.2	5.83	-8.8
5.74	-4.68	5.04	-1.7	5.88	-5.1	6.53	-19.8	6.16	-15.3
6.01	-7.71	5.33	-7.6	6.36	-14.0	6.84	-23.0	6.45	-20.0
6.33	-13.3	5.76	-11.4	6.66	-20.3	7.31	-26.8	6.9	-26.1
6.66	-14.3	6.28	-14.7	7.03	-24.3	7.64	-29.2	7.28	-30.1
6.67	-14.9	6.78	-23.7	7.39	-27.2	7.96	-28.3	7.87	-31.1
7.06	-15.0	7.26	-25.9	7.58	-31.8	8.56	-28.3	8.48	-31.4
7.38	-18.8	7.79	-30.5	7.67	-31.9	9.01	-27.3	9.15	-29.6
7.54	-19.5	8.26	-31.2	8.03	-31.3	9.57	-27.1	9.6	-28.3
8.03	-22.5	8.8	-32.2	8.65	-32.1	10.02	-26.2	9.98	-27.4
8.46	-23.4	9.26	-32.4	9.1	-30.6				
9.03	-23.5	9.76	-33.7	9.57	-29.6				
9.5	-25.0	9.98	-30.6	10.0	-28.0				
9.96	-26.6								

Table S3. Zeta potential titration values of BSA after adsorption on MSN-NH_2 in a range of BSA concentrations.

pH	BSA	pH	MSN-NH_2	pH	MSN-NH_2: BSA 1:1	pH	MSN-NH_2: BSA 1:2	pH	MSN-NH_2: BSA 1:3
3.05	26.8	2.89	32.1	3.04	31.7	2.99	27.8	3.02	28.5
3.53	22.5	3.04	32.2	3.1	26.9	3.14	30.6	3.31	26.1
4.06	16.2	3.56	31.3	3.46	28.3	3.56	24.4	3.82	20.9
4.56	12.3	3.89	29.5	3.51	23.9	4.05	18.1	4.26	16.5
5.07	4.51	4.33	28.8	4.0	19.0	4.51	12.4	4.67	11.8
5.27	1.26	4.99	26.5	4.42	13.9	5.05	5.63	5.15	3.84
5.44	0.298	5.19	26.3	4.87	7.82	5.2	2.1	5.37	0.473
5.57	-2.56	5.62	29.1	5.2	2.86	5.54	-0.428	5.5	-0.516
5.74	-4.68	5.91	28.3	5.4	1.43	5.68	-2.7	5.6	-1.92
6.01	-7.71	6.11	27.4	5.6	-1.3	6.09	-6.66	5.79	-4.59
6.33	-13.3	6.4	26.1	5.96	-6.0	6.51	-9.82	6.02	-7.02
6.66	-14.3	6.76	20.7	6.56	-9.96	7.07	-13.8	6.59	-11.2
6.67	-14.9	7.15	14.2	7.05	-13.1	7.47	-16.0	7.05	-13.6
7.06	-15.0	7.56	5.7	7.62	-17.4	8.03	-19.6	7.47	-16.4
7.38	-18.8	7.95	-1.2	8.18	-20.7	8.54	-24.2	8.01	-19.9
7.54	-19.5	8.34	-6.4	8.55	-26.0	8.99	-28.0	8.51	-24.2
8.03	-22.5	8.73	-12.5	9.01	-31.6	9.44	-32.5	9.0	-31.1
8.46	-23.4	9.13	-17.9	9.2	-33.9	9.99	-33.9	9.45	-34.6
9.03	-23.5	9.55	-21.9	9.48	-34.1			9.92	-34.9
9.5	-25.0	9.95	-25.9	9.94	-35.6				
9.96	-26.6	10.33	-27.3						
		10.97	-31.7						

Table S4. Adsorbed amount of BSA on MSN and MSN-NH_2 measured by QCM-D from data in Fig. 3.

Sample	NPs (µg/mL)	NPs adsorbed amount (ng/cm^2)	BSA (µg/mL)	BSA adsorbed amount (ng/cm^2)	S.D.	BSA/MSN[(a)]	BSA/MSN[(b)]
**MSN**	25	83	50	228	9	2	3
**MSN**	25	55	100	324	7	4	6
**MSN**	25	68	150	392	6	6	6
**MSN-NH_2**	25	78	50	246	4	2	3
**MSN-NH_2**	25	100	100	286	5	4	3
**MSN-NH_2**	25	81	150	380	12	6	5

[(a)]BSA/MSN ratio from concentrations and [(b)] calculated from adsorbed mass on the gold sensor.

Table S5. Adsorbed amount of BSA on MSN and $MSN\text{-}NH_2$ at various concentrations measured by QCM-D from data in Fig. 4.

Sample	NPs (µg/mL)	NPs adsorbed amount (ng/cm^2)	BSA (µg/mL)	BSA adsorbed amount (ng/cm^2)	S.D.	BSA/MSN[(a)]	BSA/MSN[(b)]
**MSN**	25	83	50	228	9	2	3
**MSN**	50	91	50	232	4	1	1
**MSN**	100	95	50	258	4	0.5	1
**MSN**	150	57	50	279	13	0.3	0.5
**$MSN\text{-}NH_2$**	25	78	50	246	4	2	3
**$MSN\text{-}NH_2$**	50	307	50	329	22	1	1
**$MSN\text{-}NH_2$**	100	406	50	360	16	0.5	1
**$MSN\text{-}NH_2$**	150	578	50	481	8	0.3	1

[(a)]BSA/MSN ratio from concentrations and [(b)] calculated from adsorbed mass on the gold sensor.

Estimation of the number of adsorbed BSA molecules per nanoparticle

The number of adsorbed BSA molecules per nanoparticle (Table S6) was estimated from the QCM-D adsorbed mass values (Δm). First, the surface number density of adsorbed BSA molecules (molecules·cm^{-2}) was calculated by converting the measured adsorbed mass into moles using the molecular weight of BSA (66.5 kDa) and Avogadro's number. Using the hydrodynamic radii obtained from DLS measurements (r = 72.5 nm for MSN and r = 77.5 nm for $MSN\text{-}NH_2$), the estimated number of BSA molecules per nanoparticle was then obtained by multiplying the surface number density (molecules·cm^{-2}) by the corresponding nanoparticle surface area calculated by $A_{NP}=4\pi r^2$. The reported values represent an estimate based on the external surface area of the nanoparticles. The calculation does not account for possible differences in BSA orientation effects.

Table S6. Adsorbed amount of BSA on MSN and $MSN\text{–}NH_2$ at various concentrations measured by QCM-D, together with the corresponding estimated number of BSA molecules per nanoparticle.

Sample	NPs (µg/mL)	NPs adsorbed amount (ng/cm^2)	BSA molecules per NP (× 10^3)
MSN on gold	25	69 ± 14	-
$MSN\text{-}NH_2$ on gold	25	87 ± 12	-
BSA on MSN	50	228 ± 9	1.4
BSA on $MSN\text{-}NH_2$	50	246 ± 4	1.7
BSA on MSN	100	324 ± 7	1.8
BSA on $MSN\text{-}NH_2$	100	286 ± 5	1.9
BSA on MSN	150	392 ± 6	2.3
BSA on $MSN\text{-}NH_2$	150	380 ± 12	2.6

Estimation of MSN/MSN-NH_2 surface coverage of QCM-D gold sensor

To assess the possible contribution of MSN gold interactions, the surface coverage of MSN and MSN-NH_2 on the gold sensor, prior to BSA injection, was estimated using a random sequential adsorption (RSA) model.[3,4] This model assumes spherical, non-interacting particles and a random packing limit of $\theta_{max} = 0.547$, a value obtained from Monte Carlo simulations of random sequential adsorption of hard disks on a 2D surface.[5]

Assuming spherical, non-interacting nanoparticles (NP) and monolayer adsorption, the surface coverage is:

$$\theta = \frac{\Gamma_{ads}}{M_{W,NP}} N_A \pi r^2 \qquad \text{S4}$$

Where Γ_{ads} is the adsorbed mass per unit area (ng cm^{-2}) obtained from QCM-D measurements, $M_{W,NP}$ is the NP molecular weight estimated to be 1.70×10^6 g/mol, r (nm) is the hydrodynamic radius of MSN or MSN-NH_2 from DLS measurements, and N_A is the Avogadro's number. Using r_{MSN}=72.5 nm, $r_{MSN\text{-}NH_2}$=77.5 nm and Γ_{ads} at 25 µg/mL, we obtain θ=0.3 for MSN and θ=0.5 for MSN-NH_2. At higher MSN-NH_2 concentrations (50, 100, 150 µg/mL), θ values are consistently above 0.4. At this level of surface coverage, the gold sensor is expected to be substantially screened, limiting any direct interaction between BSA and the sensor.

QCM-D adsorption of BSA on bare-gold sensor

As a control experiment, BSA adsorption was evaluated on bare gold QCM sensors in the absence of MSN or MSN–NH_2. A BSA solution (100 µg/mL in 10 mM Tris, pH 7.5) produced a mass shift of $\Delta m = 110\ ng \cdot cm^{-2}$, consistent with the formation of a protein monolayer on the gold surface. In contrast, sensors pre-coated with MSN exhibited significantly higher mass shifts, whose magnitude depended on the nanoparticle type and concentration. The time evolution of the QCM-D signal further confirms that the observed mass increase arises from BSA adsorption onto MSN rather than from interactions with the underlying gold surface.

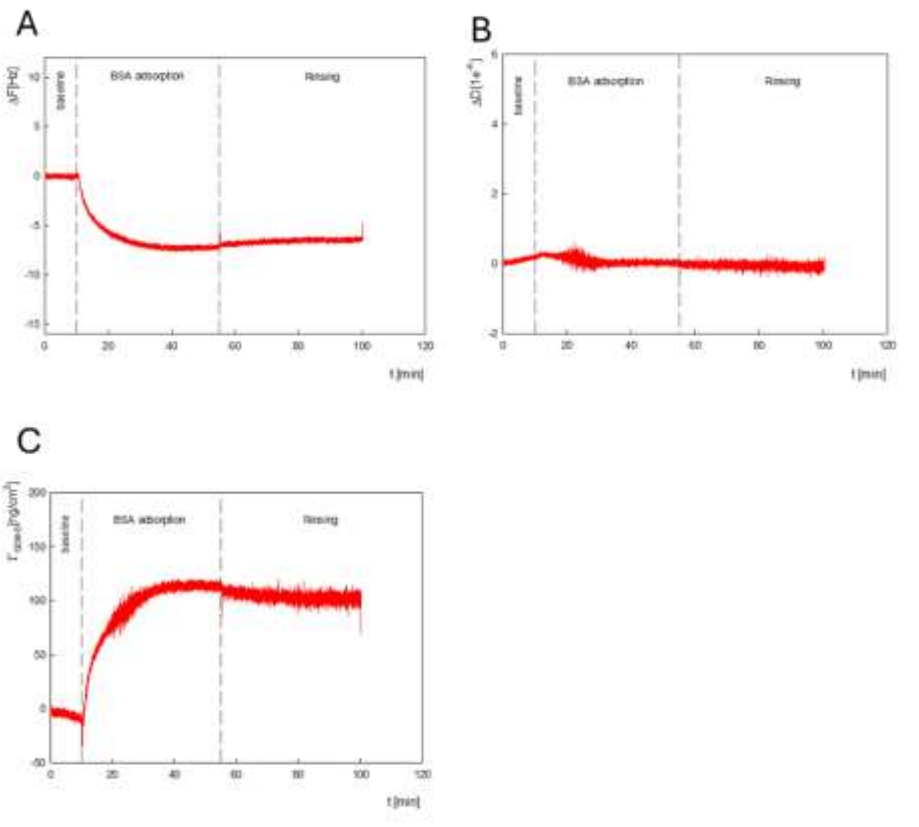

Figure S1. QCM-D measurement of BSA (µg/mL) adsorption on bare gold sensor. Frequency (A), Dissipation (B) and surface coverage (C) vs time.

References

(1) Rodahl, M.; Kasemo, B. On the Measurement of Thin Liquid Overlayers with the Quartz-Crystal Microbalance. *Sensors Actuators A Phys.* **1996**, *54* (1–3), 448–456. https://doi.org/10.1016/S0924-4247(97)80002-7.

(2) Liu, S. X.; Kim, J.-T. Application of Kevin—Voigt Model in Quantifying Whey Protein Adsorption on Polyethersulfone Using QCM-D. *JALA J. Assoc. Lab. Autom.* **2009**, *14* (4), 213–220. https://doi.org/10.1016/j.jala.2009.01.003.

(3) Kubala, P.; Batys, P.; Barbasz, J.; Weroński, P.; Cieśla, M. Random Sequential Adsorption: An Efficient Tool for Investigating the Deposition of Macromolecules and Colloidal Particles. *Adv. Colloid Interface Sci.* **2022**, *306*, 102692. https://doi.org/10.1016/j.cis.2022.102692.

(4) Jachimska, B.; Tokarczyk, K. Combining Surface Plasmon Resonance and Quartz Crystal Microbalance To Determine Hydration of Dendrimer Monolayers. *J. Phys. Chem. C* **2016**, *120* (35), 19678–19685. https://doi.org/10.1021/acs.jpcc.6b05020.

(5) Feder, J. Random Sequential Adsorption. *J. Theor. Biol.* **1980**, *87* (2), 237–254. https://doi.org/10.1016/0022-5193(80)90358-6.
